# Gender equity in scientific production in Peru and Colombia: reality or utopia in public universities?

**DOI:** 10.12688/f1000research.170019.1

**Published:** 2025-10-17

**Authors:** Félix José Colina Ysea, Nathalí Pantigoso Leython, Irene Roxana Abad Lezama, Gleny Secibel Jara Llanos, Soledad Mirella Chávez Campo, Eddy Richard Salvador Guarcaya, Myriam Esther Ortiz Padilla, Yanina Yulieth Nieto Miranda

**Affiliations:** 1 Department of Communication and Native Languages, Universidad Nacional de Educación Enrique Guzmán y Valle, Lima District, Lima Region, Peru; 2 Department of History and Geography, Universidad Nacional de Educación Enrique Guzmán y Valle, Lima District, Lima Region, Peru; 3 Department of Foreign Languages, Universidad Nacional de Educación Enrique Guzmán y Valle, Lima District, Lima Region, Peru; 4 Department of Psychology and Philosophy, Universidad Nacional de Educación Enrique Guzmán y Valle, Lima District, Lima Region, Peru; 5 Graduate Academic Department, Universidad Simón Bolívar, Barranquilla, Atlántico, Colombia

**Keywords:** Gender gap, gender equality, gender discrimination, scientific research

## Abstract

**Background:**

Within the context of research, gender equity in public universities entails significant challenges that must be addressed, leading to the restructuring of processes related to philosophy, paradigm, and management policies. This restructuring enables the conceptualization of an educational paradigm in which both men and women can cultivate their potential in an equitable manner, thereby generating scientific, technological, social, and humanistic knowledge within the scientific realm.

**Methods:**

The objective of this research was to analyze the challenges and opportunities associated with achieving gender equity in the scientific production of national universities in Peru and Colombia. A mixed-methods approach was employed to gain a global understanding of the phenomenon under study, allowing for the analysis of both quantitative and qualitative data depending on the research focus. The population and sample were defined by the national universities of Peru and Colombia, analyzing scientific production in both countries and establishing trends between male and female contributors. The technique used for quantitative data was documentary analysis of scientific production, utilizing the Scopus database for analysis. Qualitative data were collected through interviews with research managers. Instruments included a summary sheet and an interview protocol.

**Results:**

The results indicate a pronounced tendency toward higher scientific production among men across various fields of knowledge. This phenomenon exacerbates the existing gender parity gap and effectively relegates women to a secondary role within the academic landscape.

**Conclusions:**

It is concluded that national universities in Peru and Colombia must undergo restructuring processes in their organizational functions, with gender equity as a central component, in order to contribute to the co-construction of a fair and balanced society.

## Introduction

According to the most recent report of the United Nations Population Fund, ^1, 2^ women constitute nearly half of the world’s population, representing 49.7% of the global demographic. This study, as articulated in the *2023 State of the World Population,* ^3^ underscores the imperative to acknowledge the substantial presence and contribution of women within society. It emphasizes the necessity of promoting gender equity and ensuring equitable opportunities for all genders. In this regard, the International Science Council notes that the underrepresentation of women in areas such as engineering science (10%) and mathematical sciences (8%) is especially pronounced. ^4– 6^ Therefore, gender equity in scientific production continues to pose a challenge and remains a topic of growing interest and relevance in academic and social spheres.

Gender disparities in scientific publications persist over time and are evidenced by differences in the number of researchers. ^5– 8^ It is imperative to prioritize efforts that promote gender equity in academic authorship to ensure that the potential of women scientists is not overlooked. ^9^ As measured by publications and citations, ^10^ it has also been found that women are undervalued for their scientific work. These findings highlight the need for inclusive practices and targeted interventions to address gender inequity in scientific production and recognition.

In Latin America, modest advances have been observed in the realm of gender equity. For instance, there has been an increase in the participation of women in scientific research, as evidenced by the case of Mexico in the 1970s ^11^ and the establishment of the *Semillero Mujeres Investigadoras* (Women Researchers’ Seedbed; ETITC) in Colombia. ^12^ These developments have facilitated the presence of women across various disciplines, particularly in research. However, significant challenges remain regarding the equal participation of men and women in terms of scientific production. The gender gap in academia and research is a multifaceted phenomenon that extends beyond simple numerical representation. Women’s participation continues to be influenced by structural variables. ^13^ Several factors contribute to this phenomenon. First is the academic offerings, specifically women’s enrollment in STEM and science programs. Second are research policies, including calls for proposals, the formation of evaluation committees, organizational culture, inclusive funding, and the distribution of incentives and/or recognition. Third is the researcher’s profile, which encompasses economic income, geographic location, age, marital status, family responsibilities, educational level, and field of study. ^14^

In countries such as Peru and Colombia, has gender equity truly been achieved in this field, or does it remain a utopia? A multitude of studies have demonstrated that women are underrepresented in both publication and citation rates across various scientific disciplines. ^15^ This underrepresentation is also evident in the field of academic research, where women are less likely to be first authors, often occupying a more marginal position. ^16^

In the specific context of Peru and Colombia, disparities in the participation and representation of women in scientific production persist. Although policies and programs have been implemented to promote gender equality in academia, much remains to be done to address the underlying challenges and create a truly inclusive and equitable environment for all researchers, regardless of gender. In Peru, women constitute only 30% of researchers. Furthermore, among all authors listed as first authors in research papers, only 26% are women. ^17^ In Colombia, women represent just 38% of researchers. A recent study revealed that there are 6,411 female scientists in the country. This figure is particularly noteworthy in light of the increasing discrepancy in publication output between senior researchers, those with greater seniority or experience, and researchers with doctoral degrees. ^18^ This suggests that even after obtaining advanced degrees and entering the workforce, women continue to be underrepresented in academic output compared to men. A gender gap of 5.8% is evident among emeritus researchers, defined as those over 65 years of age who have made significant contributions in their respective fields. This disparity, observed between 2015 and 2021, indicates that male researchers are more likely to attain emeritus status than their female counterparts. Such inequities can be attributed to systemic barriers encountered by women rooted in pervasive discrimination. ^19^ In other words, access to graduate education and labor integration alone are not sufficient to ensure equal representation of women in research. Researchers from Peru and Colombia have made important contributions across various fields.

In Peru, studies have demonstrated that female scholars specializing in psychology produce a lower volume of scholarly work than their male counterparts. Additionally, women scholars tend to collaborate more frequently with male counterparts and focus their research on clinical and health-related topics. ^20^ In Colombia, a generalized gender disparity in scientific publications has been observed, with high-level female researchers and those holding doctoral degrees showing the lowest levels of editorial participation. ^7^ Furthermore, a gender gap in scientific production has been documented among Peruvian physicians, with observable factors such as years of medical practice, researcher accreditation, and academic status contributing to the disparity. ^21^ These findings underscore the need to address the underrepresentation of women in scientific research and to promote equity, diversity, and inclusion in the scientific workforce in both Peru and Colombia. Therefore, the research question guiding this study is: “What are the challenges and opportunities that will contribute to achieving gender equity in the scientific production of national universities in Peru and Colombia?” The general objective of the study was to analyze the challenges and opportunities in achieving gender equity in the scientific production of national universities in both countries.

### Background and theoretical basis of the study

At the global level, research conducted for the Organization of Ibero-American States (OCTS-OEI) on scientific production in gender studies, based on journals indexed in Web of Science (WOS) for the 2008–2018 period, shows that women tend to focus on issues related to gender, politics, and violence, while men more often research topics associated with medicine, psychology, and statistics. ^22^ In this context, a study carried out by Universidad Tarapacá y Talca in Chile on gender gaps in scientific production, using a bibliometric analysis of published articles and the Scival and Dataciencia databases, revealed that women publish fewer articles, thus marking a gap in contrast to their male peers, who are more productive. ^23^ In Brazil, an analysis of publications from the Foundation for the Support of Scientific Research of the State of São Paulo (FAPESP) showed a predominance of male authorship in areas such as Social and Physical Sciences. By contrast, greater gender balance was observed in areas such as Life and Health Sciences. Furthermore, a decline in female authorship after 2021 was noted, which could be explained by the impact of the COVID-19 pandemic. ^24^ In Peru, a bibliometric study based on data provided by CONCYTEC identified that 68.1% of publications were authored by men and 31.9% by women. ^25^ In Colombia, Social Network Analysis (SNA) was used in research on productivity and gender equality within scientific support groups with free and varied information. Data were obtained from the GrupLAC application of the SCIENTI platform, part of the Ministry of Science, Technology and Innovation of Colombia (MINCIENCIAS). This analysis characterized the gender and scientific production (articles and books) of researchers participating in the research process. It was concluded that, with respect to the scientific production of research groups and teachers, men participated at higher rates than women. The study highlighted the need to improve the process of scientific collaboration. ^26^

### Theoretical review of the gender equity variable

In order to elucidate the concept of gender equity, it is imperative to first understand the definition of the term “gender” from an anthropological perspective. Gender is a social construct that serves to differentiate between what is considered sexually feminine and what is masculine. ^27^ Like any human institution, it is socially instituted, ^28^ a learned behavior influenced by society and an analytical category that helps explain behavioral differences between men and women. In recent decades, this category has been shaped by the social sciences, particularly through feminist theoretical frameworks, which initially promoted the analysis of men and women and the relations of inequality and oppression between them. ^29^

It is crucial to distinguish between the terms “equity” and “equality.” Equality is an abstraction that is difficult to materialize, as society does not contain identical phenomena; each one is unique in its nature. ^30^ In this sense, speaking of equal opportunities between men and women can be considered utopian, given their inherent differences. Therefore, it becomes necessary to implement compensatory actions to move beyond simple equality, i.e., to provide more to those who have less. The term “equity” is closely associated with the concepts of equality and social justice. It involves a set of actions aimed at addressing systemic issues and promoting societal transformation toward a more just and equitable environment. ^31^ In this context, the concept of equity becomes even more relevant. Equity does not seek absolute equality but rather aims to ensure that everyone has the same opportunities to fully develop, regardless of their gender.

The fifth goal of the United Nations 2030 Agenda calls for gender equality and the empowerment of all women and girls in every sphere of human life. This objective is considered a fundamental and essential right for a prosperous and equitable world. ^32^ However, the *2023 Sustainable Development Goals Report* indicates that progress toward gender equality indicators has been insufficient and remains far from the targets set for 2030. ^32^ It is clear that much remains to be done to achieve gender equity in our society. The struggles for equity and emancipation are ongoing, and they are now taking place in decision-making spheres where scales, indicators, and accreditations are established. These can be used to measure performance, salaries, inputs, and even roles, with the aim of reflecting equity. ^33^

The gender gap in Latin America and the Caribbean is a highly relevant issue that has gained increasing attention in recent years. It refers to the existing inequalities between men and women in terms of access to resources, opportunities, and involvement in different areas of life. Studies by the Economic Commission for Latin America and the Caribbean (ECLAC) show that Latin America and the Caribbean is the most unequal region in the world in terms of gender equality, considering the various factors that affect women’s participation. ^2^ Although women’s participation in the labor force has increased, gender wage gaps and segregation in occupational roles still persist. It is common for women to work in lower-paying jobs with limited opportunities for accessing leadership and decision-making positions in the workplace. ^34^

Addressing gender inequality is essential due to its adverse effects on human welfare, social justice, and sustainable development. Moreover, gender inequality hampers the achievement of development goals and the formation of just and harmonious societies. ^35^ A significant gender gap also exists in the scientific field: women tend to publish fewer papers, are cited less often, are nominated for fewer awards, and receive less funding. ^2^ A multitude of factors have been identified to explain the gender disparities observed in the scientific realm. Among these factors is the tendency of women to prioritize familial responsibilities over academic pursuits. Additionally, the temporal dissonance between academic obligations and social interactions, particularly among women who may hesitate to negotiate for promotions, has been identified as a contributing factor. Furthermore, the lack of tangible recognition for achievements may serve as a deterrent, possibly indicating a potential lack of ambition and self-limitation among women. ^36^

In this sense, it is important to define the variable. Gender equality is a principle aimed at guaranteeing equal opportunities, rights, and benefits for all individuals, regardless of gender. ^37^ Gender equality means fairness and equity, wherein men and women are treated according to their respective needs. This involves ensuring equal access to resources and opportunities without discrimination. ^38^ Gender equality implies the fair distribution of resources and opportunities between men and women in society, ensuring that systemic barriers are removed and equitable outcomes are promoted. Gender-specific needs must be recognized and addressed. ^39^

### Theoretical review of the scientific production variable

It is acknowledged that science is a social construct, subject to the norms and standards of the scientific community. This understanding leads to the recognition that scientific knowledge is a socially produced, legitimized, and stabilized process. It is also recognized that the stability of this knowledge is temporary and by no means definitive, due to its inherent dynamics of re-conceptualization based on contextual needs. This process contributes to the production of new knowledge in science, aiming to validate or test prevailing theories in a specific field. In this sense, scientific research is the result of a rigorous, systematic, and creative process that seeks to find answers to humanity’s problems and to achieve new forms of human knowledge. It is generally accepted that research processes underlie the reality of universities, institutions, and research centers, as well as the research policies issued by each country. Research thus constitutes the essence and identity of each institution.

From this point of view, scientific production represents the intellectual contribution made by researchers, who communicate the results of their studies and contribute to the development of science. ^40^ Within the university context, scientific production emerges as a catalyst for social transformation, driven by the potential for a comprehensive and interdisciplinary perspective. ^41^ It functions as a dynamic axis in the edifice of novel knowledge and the understanding of reality, arising from the research and innovation efforts of university professors. These efforts manifest in a myriad of products, including theses, projects, patents, articles, and scientific conferences, each of which addresses the resolution of a specific problem. Furthermore, shifts toward the digital environment have influenced scientific production on a global scale, ^42^ highlighting several developments. These include the transition of scientific journals from print to digital formats and the emergence of open access, which has fostered the dissemination of research findings without the constraints of economic, technological, or legal barriers.

Scientific production is the tangible manifestation of generated knowledge, which goes beyond a simple collection of documents stored in an information institution. It encompasses all the academic and scientific activities undertaken by a researcher. ^40^ This phenomenon is closely linked to many events in which people are involved daily. The evaluation of scientific production, based on the results of research and innovation outputs, is not a recent practice in many disciplines. However, its study has intensified and become more systematized in the past two decades, underscoring its growing importance in academic and scientific spheres.

Productivity is defined as the total volume of research generated by scientists, primarily assessed through the number of publications authored by an individual scientist, an institution, or a specific nation. ^43^ This measure is fundamental to understanding and evaluating the impact and contribution of researchers and academic entities to the global advancement of knowledge and science. Scientific productivity refers to the body of publications in scientific journals, whether by authorship and/or co-authorship. ^44^ Finally, scientific production is the process of disseminating the results of research through information channels, such as scientific articles published in indexed journals. ^45^ This process is essential for the communication and advancement of scientific knowledge, allowing researchers’ findings to be evaluated, replicated, and applied by the global academic and professional community.

The most frequent sources of scientific production include, first, scientific publications and texts, periodicals containing studies and results of original research, as well as complete works or specific sections authored by experts, providing comprehensive or detailed perspectives on specific topics. Second, scientific events, where researchers present and discuss their work with other experts in the field. Third, undergraduate and graduate theses, research reports, and unpublished papers resulting from academic research and research work conducted as part of advanced degree requirements, such as master’s and doctoral programs, as well as institutional repositories and databases, which are online platforms where academic and research institutions store and share their scientific work. Fourth, patents, which are documents describing new inventions or technological developments and granting exclusive exploitation rights to inventors. Fifth, scientific exhibitions, which include compilations of papers and presentations delivered at scientific congresses that highlight the latest findings and developments in specific fields. Finally, normative documents and methodological guidelines for scientific dissemination, publications aimed at broader audiences that explain and communicate scientific advances in an accessible manner. ^40^ These sources are essential for the dissemination and validation of scientific knowledge, enabling other researchers to review, replicate, and build upon prior work.

## Methods

The mixed approach represents the collection of quantitative and qualitative data that are harmoniously articulated to obtain a global and interpretative vision of the phenomenon under study. In this sense, the research adopted a mixed approach to understand the realities associated with the variables *gender equity* and *scientific production* in national universities in Peru and Colombia, leading to the generation of both numerical and comprehensive results within an interpretative vision. Likewise, the method was analytical, allowing for an understanding of reality from both an objective and subjective perspective in interaction with the social context. ^46^ Consequently, this approach is regarded as a foundational framework for comprehending and interpreting the phenomenon of gender equity and scientific production in national universities in Peru and Colombia.

The mixed-methods design is conceived as the combination of quantitative and qualitative data; its nature is dynamic, interactive, and reflexive, as it engages directly with the reality of the phenomenon under study. ^46^ In this sense, the design determines the path or activities that the researcher must undertake to achieve the study’s objectives. Therefore, the approach adopted in this study is consistent with a dynamic, interactive, and reflexive design. This design was employed to analyze gender equity and scientific production in national universities in Peru and Colombia.

*Population* refers to a set with similar characteristics, represented by people, organisms, objects, or animals, depending on the nature of the phenomenon under study. ^47^ The research included fifty (50) national universities accredited by the National Superintendence of University Education (SUNEDU, for its Spanish acronym) in Peru, as well as thirty-four (34) national universities in Colombia, accredited by the Sub-Directorate for Quality Assurance in Higher Education through the Quality Assurance System in Higher Education (SACES, for its Spanish acronym). The population thus consisted of 84 national universities from Peru and Colombia. In addition, six (6) research professors or research managers with a minimum of five (5) years of experience at the university level were interviewed to understand perceptions of gender equity in scientific production in both countries. Participants were selected intentionally and ensuring representativeness in each country (three Peruvians and three Colombians). They were initially contacted by telephone and later by email.

The study was approached through integrative phases to gain a deeper understanding of the phenomenon under investigation. The *exploratory phase* enabled an examination of the variables under study, contextualizing the phenomenon within the realities of national universities in Peru and Colombia, along with a review of the theoretical foundations of the variables. Consequently, the *descriptive phase* entailed the formulation of a work plan or research design, which served as a guide for researchers in achieving their objectives. During this phase, the instruments to be used for both quantitative and qualitative data collection were identified. As a final result, conclusions and recommendations were generated for subsequent dissemination of the findings in scientific journals.

To analyze trends in scientific production in national universities in Peru and Colombia from a gender perspective, data were reviewed from institutions responsible for scientific development and research in each country—specifically, the Ministry of Science of Colombia ( https://minciencias.gov.co/la-ciencia-en-cifras/produccion) and the National Council for Science, Technology and Innovation of Peru ( https://servicio-renacyt.concytec.gob.pe/busqueda-de-investigadores/) were reviewed. However, it should be noted that the data for Colombia were only updated through 2021, which precludes an entirely equitable comparative analysis. Therefore, the analysis proceeded using data from the Scopus database. Filters were applied to isolate scientific production by country, taking into consideration the time range from 2022 to October 2024, selecting only documents with a final publication status, and limiting participation to affiliations with public universities. In Peru, 5,075 publications were identified whose first author is affiliated with a public university, while in Colombia, 14,747 such publications were identified.

To establish the distribution by gender, a binary classification model (male and female) was used, as the database did not include this criterion. This is a recurrent methodology in scientometrics ^24^; however, its limitations should be noted, as the variety of names and the existence of gender-neutral names may result in erroneous assignments in gender classification.

For the inference process, the artificial intelligence (AI) Data Analysis Report AI developed by OpenAI was selected, employing a machine learning model. The methodology began with the preparation of the dataset and the identification of the names of the primary authors of each publication. Subsequently, a supervised machine learning model was used to classify the names based on prevalent patterns associated with male and female names. Ultimately, the model was implemented to categorize each publication. This process enabled the establishment of gender classification for the entire Colombian sample. However, in the case of the Peruvian data, 337 publications could not be categorized due to the absence of sufficient information—specifically, the initial letter of the name—which precluded their classification.

The interviews were conducted by mutual agreement between the researchers and key informants, who were asked for and provided their written informed consent. The interviews were held via Google Meet, which allowed for full recording. An interview script was used as an instrument to facilitate understanding of the study phenomenon. Once the interviews were conducted, they were transcribed, and the document was sent to each of the interviewees for validation. Bubbl.us, ^48^ a tool that enabled the structuring and connection of emerging categories, was used for the development of the category network.

## Results

This section elucidates the information analyzed based on the variables of gender equity and scientific production. It is worth mentioning that the research adopted a mixed approach, offering both quantitative and qualitative results. Each is presented below.

### Results of the quantitative analysis

To analyze the trend of scientific production in national universities in Peru and Colombia with respect to gender, the institutions responsible for the development of science and research in each country—the Ministry of Science of Colombia ( https://minciencias.gov.co/la-ciencia-en-cifras/produccion) and the National Council for Science, Technology and Innovation in Peru ( https://servicio-renacyt.concytec.gob.pe/busqueda-de-investigadores/)—were reviewed. In addition, data obtained from the Scopus database were used. Filters or criteria were applied to limit scientific production by country, considering the time range from 2022 to October 2024, final publication status, and participation limited to affiliations associated with public universities.

Table 1 shows that Colombia’s scientific production has been greater than that of Peru, nearly tripling it. Regarding classification by gender, the trend in both countries shows that men outnumber women. It should also be noted that the gap is wider in Peru than in Colombia.

**Table 1.** Scientific production by gender (2022–2024) in public universities of Peru and Colombia.

Gender	Female	%	Male	%	Unknown	%	Total
Peru	1782	35.1%	2956	58.2%	337	6.6%	5076
Colombia	6416	43.5%	8331	56.5%	0	0.0%	14747

Note: Statistics by gender.

Table 2 shows that in both countries, men outnumber women in most types of publications, with the exception of the “Note” publication type in Colombia, where women slightly outnumber men by a margin of 0.5%. However, Colombia has a higher proportion of women than Peru in several categories.

**Table 2.** Scientific production by publication type (2022–2024) in public universities of Peru and Colombia.

Country	Publication type	Female	%	Male	%	Total
Peru	Article	1458	38.9%	2283	61.1%	3742
	Book	0	0.0%	1	100.0%	2
	Book chapter	20	34.9%	37	65.1%	58
	Presentation	205	31.0%	456	69.0%	662
	Data paper	2	100.0%	0	0.0%	3
	Editorial	4	19.0%	17	81.0%	22
	Erratum	1	20.0%	4	80.0%	6
	Letter	21	31.8%	45	68.2%	67
	Note	5	38.5%	8	61.5%	14
	Retracted	0	0.0%	0	0.0%	0
	Review	65	38.5%	104	61.5%	170
	Short survey	1	50.0%	1	50.0%	3
Colombia	Article	5244	43.8%	6699	56.2%	11944
	Book	3	33.3%	6	66.7%	10
	Book chapter	132	43.0%	175	57.0%	308
	Presentation	333	38.9%	523	61.1%	857
	Data paper	13	36.1%	23	63.9%	37
	Editorial	74	35.4%	134	64.6%	209
	Letter	130	50.0%	130	50.0%	261
	Erratum	32	46.4%	37	53.6%	70
	Note	56	50.5%	55	49.5%	112
	Retracted	0	0	1	100%	1
	Review	454	45.7%	539	54.3%	993
	Short survey	9	50%	9	50%	18

Note: Statistics by scientific production.

Table 3 shows a general trend in which men dominate publication across all academic areas analyzed, especially in *Natural Sciences* and *Engineering and Technology*, where female representation is notably low in both Peru and Colombia. Areas such as *Social Sciences* and *Medical and Health Sciences* show a smaller gap compared to the other fields.

**Table 3.** Scientific production by OECD area (2022–2024) in public universities of Peru and Colombia.

Country	OECD Area	Female	%	Male	%	Total
Peru	Agricultural Sciences	21	30.9%	47	69.1%	69
	Medical and Health Sciences	158	44.1%	200	55.9%	359
	Natural Sciences	1331	37.7%	2196	62.3%	3528
	Social Sciences	28	38.9%	69	71.1%	98.1
	Humanities	116	35.7%	209	64.3%	326
	Engineering and Technology	128	35.3%	235	64.7%	364
Colombia	Agricultural Sciences	693	42.5%	937	57.5%	1631
	Medical and Health Sciences	2201	44.7%	2723	55.3%	4925
	Natural Sciences	719	43.7%	928	56.3%	1648
	Social Sciences	878	45.0%	1074	55.0%	1953
	Humanities	690	44.0%	880	56.1%	1571
	Engineering and Technology	1235	40.8%	1789	59.2%	3025

Note: Statistics by area of knowledge.

### Results of the qualitative analysis

Six (6) interviews were conducted in June and July 2024 with university professors experienced in research and/or research management for the purposes of qualitative analysis. The semi-structured interviews were based on an interview script related to the topic of the study and were conducted through Google Meet, which served as an instrument to accurately record the information provided by key informants. Once the information was gathered, the interviews were transcribed verbatim for analysis. This process included microanalysis, or content analysis, line by line and word by word, of each information protocol. In addition, the method of constant comparison between each of the dimensions and properties was employed simultaneously, ensuring consistency. Furthermore, the qualitative results are presented in natural thematic units, highlighting the opportunities within the management of national universities in Peru and Colombia to promote gender equity in scientific production. Each interview was coded as Interview No. 1 through Interview No. 6 to protect the personal data of the interviewees. The results of each thematic unit are presented in Table 4 below.

**Table 4.** Opportunities in university management for gender equity in scientific production.

Thematic unit: Opportunities for the management of national universities in Peru and Colombia to achieve gender equity in scientific production	
Interviewees No. 1−6	
Comments	Interpretation
Interviewee 1: “It is necessary to have research policies that lead to improved scientific production and gender equity.” “Yes, those conditions do exist, but it also depends on our vision and ability to look at our reality and contribute to it.” “Therefore, implementing that policy is the way forward at this point. Here, there is an early childhood law that guarantees children access to education, programs, and inclusion.” “Right now, there is a concept here called the quadruple helix, where civil society, organizations, and public and private entities come together to propose new actions.” Interviewee 2: “There must be a paradigm shift in university management and, with this, a change in research procedures.” “I believe that creating opportunities for all individuals and taking the time to consider the competencies for which they are trained and preparing themselves could potentially eliminate gender gaps. We are the ones who are creating the gaps.” “I believe that the opportunity should be available to everyone, and yes, women have been greatly affected. I thank history, I thank the women who came before me, I thank the organized minority groups, but we are no longer a minority.” “I believe that it has to do with empowerment and access.” Interviewee 3: “Opportunities are there. What we need is a new vision of managing research, giving opportunities to women.” “In general, Peruvian universities do not invest heavily in research; there are no major research development policies. The research courses included in university curricula are imposed by the regulator.” “So, really, to think that universities have strong research policies and that, unfortunately, women are discriminated against in them is not entirely accurate, because in general, university research policies are weak. So, let’s say that in that situation, universities are already interested in issues related to rankings, which basically includes women, right?” Interviewee 4: “We need to be given opportunities to generate changes in research.” “Maybe there are some policies that give women flexibility to continue their work or research, but in these processes, for example, maternity or pregnancy, we need to give them that flexibility of virtuality. The researcher’s work, therefore, does not require her to be in the field.” “Evaluating the person more by their scientific productivity—articles, papers—than by the hours sitting at a desk.” Interviewee 5: “Universities must understand the importance of women and their capabilities in research management.” “So this also has to be considered when working on gender policies, because sciences and structures are not the same. Every policy has to be thought about at both the big-picture level and the level of differences. Maybe there are more women than men in some subjects and fewer in others. It’s more about whether I should give extra points to one or the other because I want to seek equality, and the same should apply to all.” Interviewee 6: “We must work hand in hand with both women and men in conditions of equality, as this leads to maintaining an increasingly fair and equitable society, eliminating social stereotypes.” “Give more opportunities to women. Provide opportunities to promote projects funded by the state, by CONCYTEC, and give women the opportunity to do research on this topic and introduce proposals to improve reduction. Problems are not solved by a simple guideline but by constant participation and research. As part of this, identify the gaps. In other words, what are the real causes or factors that create this gap?”	To generate substantial changes in universities in terms of gender equity in scientific production, it is necessary to establish research policies that are consistent with the current context, providing equal conditions for both men and women. Opportunities must give equal opportunities that lead to the inclusion, integrity, and participation of men and women as equals. A paradigm shift is needed in university and research management, where both men and women are integrated to lead to sustainable transformations contributing to the development of an equitable society. Under this vision, universities must guarantee conditions of equality so that both men and women can develop their professional and research skills within spaces of participation, respect, and tolerance, contributing to the development of an increasingly fair and equitable society. Therefore, opportunities will always be latent; it is necessary for men and women to assume those opportunities and crystallize them in a university management that bets on balance and equal conditions for all. Likewise, the opportunities that universities should assume must be accompanied by research policies coherent with the social context and the vision of humankind as a social subject, leading to integrated processes and generating spaces of equality so that both men and women can develop their knowledge. Therefore, there is an urgent need for a change in the way national universities in Peru and Colombia are managed. This change must include women playing a very important role in research, with men helping them. This would help research develop. This requires providing the necessary conditions in terms of research policies, as well as adequate processes and procedures that lead to systemic change in universities. Furthermore, research policies must be understood by all, reflected as core axes in all academic processes, with the true spirit of research emerging in each of the educational actors involved in the process. That is, research as a formative process, but at the same time, research as a scientific process. In this way, scientific research must come along with popular knowledge, as a process of integration where both emerge to give a different vision in the approach to social phenomena. Therefore, universities must have good research policies that, on the one hand, should create research processes and, on the other, respond to the social needs around us.

Note: Excerpt from the interview with researchers.

**Figure 1.**
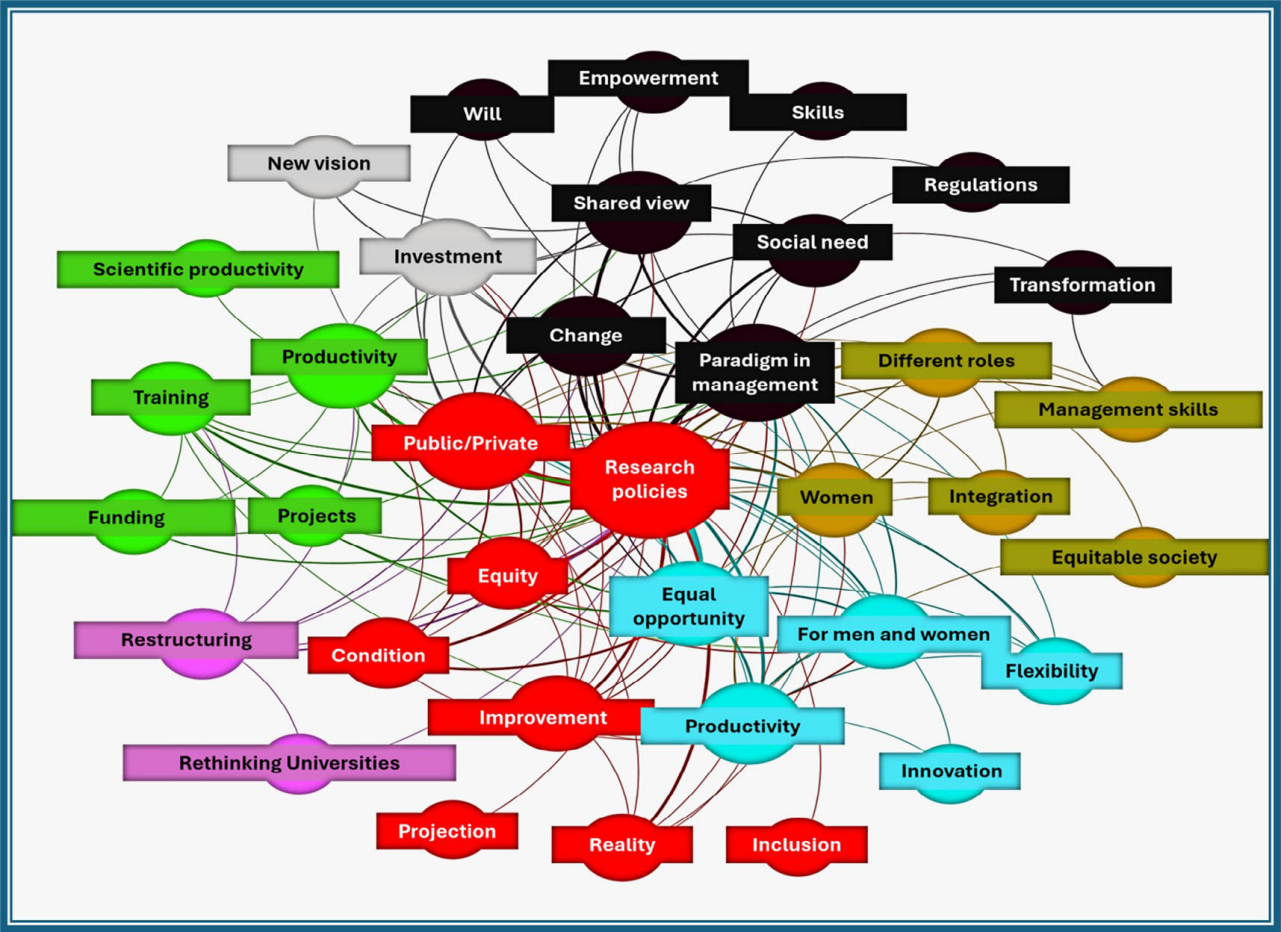
Semantic network of university management for gender equity in scientific production.

The Figure 1 illustrates the semantic network obtained from the coding and constant comparison of the six interviews conducted with university professors in Peru and Colombia. Central categories such as Research policies, Equal opportunity, and Equity are interconnected with themes related to productivity, innovation, inclusion, and institutional management. The network highlights the role of public and private sectors, the importance of investment and training, and the relevance of management skills and regulatory frameworks. Together, these elements reflect the opportunities and challenges for promoting gender equity in scientific production within national universities in both countries.

## Discussion

In the context of research, gender equity is a multifaceted, intricate, and systemic concept that must be comprehended from diverse social perspectives. It entails the interweaving of relationships with other concepts, thereby contributing to the development of an equitable society. In this sense, men and women serve as the central axis of social transformation. ^27^ Consequently, gender equity at the university level should be regarded as a pivotal component in the joint construction of the concept of man as a social entity. This entails the establishment of spaces that facilitate the development of competencies among both women and men within an environment characterized by respect, participation, tolerance, and contribution to the sustainable and inclusive advancement of society. ^22^ On this basis, it is worthwhile to examine the metrics of scientific production at national universities in Peru and Colombia, taking into account the gender criterion. The study revealed a predominance of male over female scientific production in both Peru and Colombia. These results show that, although efforts are being made to achieve gender equity within universities, it seems to be limited by the mindset of the management itself, entrenched in a traditional paradigmatic vision and even the same stereotypes present in society, as a limiting factor that considerably affects the progress of gender equity in universities. It is necessary to generate changes in their functions, with gender equity as the main axis. ^22^

According to, ^23^ equal opportunities must be given at universities so that both men and women can develop their competencies in accordance with their vocation for service, promoting the development of an inclusive society and the formation of a social subject who can understand differences but, at the same time, has the ability to integrate points of disagreement, contributing to the global understanding of their own social context. Therefore, the social subject to be formed must internalize the vision of their own reality, understand their socio-historical context, and allow spaces for inclusion where both men and women can develop their professional, personal, and social competencies.

In understanding the trend of gender equity in scientific production in the national universities of Peru and Colombia, a very marked tendency of male dominance in scientific production can be observed, since men have historically led research projects, leaving women to perform office activities, an aspect that has limited scientific production by women. ^25^

In addition, the scientific production of men places them in an important plane of analysis, since they often lead the research and are the main or corresponding authors, whereas women are relegated to third, fourth, or other co-authorship positions. ^26^ This reality calls for changes in research management, as it is necessary to provide equitable conditions for both men and women to lead research projects. There is an urgent need for a renewal in the paradigmatic vision of scientific production, since women have been gaining important spaces in the country’s scientific, technological, and social development.

Under this vision, it is necessary to offer opportunities for equity in scientific production, leading to the generation of scientific indicators that allow for the evaluation of production metrics, in addition to trends or variables for comparison, giving rise to a situational analysis of the role of women in science. ^35^ Hence, science itself must become a voice of inclusion, of transcendental change, and of equitable conditions, contributing to the formation of a fair and just society.

Regarding the perception of research professors about the conditions for gender inclusion in research development in national universities from Peru and Colombia, the results show that the process of gender inclusion is slow. In other words, there is no clear intention from the universities to generate substantial modifications to change the panorama of the underrepresentation of women in the different areas of scientific knowledge.

Universities, as spaces for divergent thinking and key actors in the socio-productive development of the nation, must carry out internal and external situational analyses, ^37^ leading to the reformulation of their research policies and the generation of changes not only in the drafting of documents but also in the organizational culture and in the management of the institution’s human talent. Diagnosing the gender distribution of the scientific production at each university should constitute the starting point.

Universities must initiate processes to reformulate their policies in order to guarantee gender equity, ^38^ leading them to disassociate themselves from linear, utopian, and traditional thinking, which limits the vision of change held by university management. Providing women with spaces for the meaningful co-construction of an increasingly plural society is therefore necessary, where men and women join forces in the academic formation of the new social subject that society demands.

Moreover, the absence of gender equity conditions in universities should not be reflected in inert documents but rather in the actions of every collaborator, providing equal conditions for all and ensuring productivity in each of the functions to be performed. ^38^ Therefore, it is necessary to generate substantial changes in the organizational structure of universities, in the paradigms that sustain them, and in the mental framework of their human talent.

Humanization processes are imperative in academic institutions to comprehend individual as well as collective aspects, thereby facilitating the integration of perspectives into the organization’s productive development and the cultivation of human talent.

Furthermore, with respect to the challenges and opportunities inherent in the management of national universities in Peru and Colombia, it is imperative to achieve gender equity in scientific production. To this end, universities must make substantial changes to their paradigms and to their human capital, as a multifaceted process that facilitates the accumulation of scientific knowledge.

Urgent academic-administrative restructuring at universities is required, one that transcends bureaucratic procedures and focuses on the human essence, the socio-historical context, and the value of the self as a historical subject. This triad must be internalized as a transformation process reflected in the actions of each university actor, providing conditions of equity in which men and women can carry out their academic and administrative responsibilities, thereby making a substantial contribution to the development of both the university and society. Universities must interpret their socio-historical context, the evolution of human beings in their formative processes, and the construction of an inclusive society, thus generating opportunities for both men and women to develop their competencies in a balanced way. ^41^

The transformation should extend beyond the research processes of universities, ^42^ wherein research departments and vice-rectorates develop policies in accordance with prevailing circumstances, resulting in a shift in the paradigmatic vision of research direction. This transformation must internalize the value of women and provide ideal conditions for their significant contributions to the country’s scientific development. This implies a systemic complement, not an exclusion of men, but rather their integration into a systemic vision that enables both men and women to generate substantial changes in the paradigmatic vision of how research is approached.

Therefore, research directorates and vice-rectors’ offices must elaborate research policies and create procedures and norms that offer a range of opportunities so that both men and women can develop their full potential. ^45^ This leads to an understanding of research as both a training process and a research process, where men and women are integrated in a balanced manner for the development of research projects that are reflected in the scientific production of universities. Thus, it can be concluded that women will not be granted privilege solely on the basis of their own circumstances. Rather, it is a process of integration in which both men and women are given the same opportunities for research development.

## Ethical considerations

The research project was reviewed by the Ethics Committee of the Universidad Nacional de Educación Enrique Guzmán y Valle and approved with Resolution No. 1077-2024-R-UNE, on March 27, 2024. The committee verified that the research respected the dignity, identity, well-being, and data privacy of the study sample, without altering the human condition. It should be clarified that the data published in Scopus were used solely for AI analysis, and no additional information about the researchers was disclosed; therefore, ethical risk was minimized and privacy was protected. Similarly, the participants in the interview sample were informed about the research process and provided their voluntary participation through written informed consent. The personal information of this study sample was kept strictly confidential by the researchers.

## Data Availability

Figshare. Research Materials and Data from the Gender Equity in Scientific Production Project. https://doi.org/10.6084/m9.figshare.30168850.v2. ^49^ This project contains the following underlying data:
• Transcript of interviewee responses • Informed consent form template • Interview script • Data on scientific output from public universities in Peru • Data on scientific output from public universities in Colombia Data are available under the terms of the Creative Commons Attribution 4.0 International license (CC BY 4.0). Figshare. Supplementary Material – COREQ Checklist (Gender Equity Study). https://doi.org/10.6084/m9.figshare.29997016. ^50^ This project contains the following underlying data:
• COREQ Checklist Data are available under the terms of the Creative Commons Attribution 4.0 International license (CC BY 4.0).
