# Supplementary material for: Gender equity in scientific production in Peru and Colombia: reality or utopia in public universities?
Source: F1000Res. 2025 Oct 17;14:1128. [Version 1] doi: 10.12688/f1000research.170019.1 (PMC12853017; doi:10.12688/f1000research.170019.1)
Supplement: Peer review history

# Reviewer report: Reviewer response for version 1

**DOI:** 10.5256/f1000research.187430.r449961

**Published:** 2026-01-28

**Authors:** Luis Marcelo Olivos Jimenez

**Affiliations:** 1 Universidad Cesar Vallejo, Chimbote, Ancash, Peru

The article focuses on the gender gap in scientific output at state universities in Peru and Colombia. It uses a mixed approach, with quantitative data on scientific output and interviews with university research managers. It concludes that there is a significant disparity and predominance of men in scientific production. It also shows that gender equality has not yet been achieved and that institutional changes are needed at the structural and cultural levels to make universities more equitable and inclusive.

 The topic addressed is extremely important and relevant, especially in the context of gender equality policies in higher education. The mixed design is relevant, as it allowed for the collection of a wide range of publications indexed in Scopus from 2022 to 2024 and the exploration of perceptions regarding gender equality. The classification of authorship by gender used a common approach in bibliometric studies, assigning sex based on proper names, but this is not foolproof, as it does not take into account possible mixed or ambiguous gender names. However, this does not invalidate the findings. It should be noted that this was also pointed out by the authors. The quantitative results show existing gender gaps, with male output exceeding female output in both countries, with Peru having the widest gap. This trend persists in most types of publications and areas of knowledge. In terms of qualitative results, the perceptions of the interviewees are consistent with the quantitative results. They also propose the need for explicit institutional policies on gender equality and paradigm shifts in the academic management of universities that promote truly effective inclusion of women in scientific research. The results are linked to the UN Sustainable Development Goals and the global theoretical support for gender equality.

 In conclusion, the study fulfills its stated objective of analyzing gender equality trends in scientific production at public universities in Peru and Colombia. The mixed methodological approach provides a comprehensive understanding of the phenomenon. Both qualitative and quantitative dimensions are clearly identified. Despite some methodological limitations, these do not compromise the overall conclusions of the study.

Is the work clearly and accurately presented and does it cite the current literature?

Yes

If applicable, is the statistical analysis and its interpretation appropriate?

Yes

Are all the source data underlying the results available to ensure full reproducibility?

Yes

Is the study design appropriate and is the work technically sound?

Yes

Are the conclusions drawn adequately supported by the results?

Yes

Are sufficient details of methods and analysis provided to allow replication by others?

Yes

Reviewer Expertise:

Research management and policy in higher education; scientific production and bibliometrics; gender equity in research; university research evaluation; science, technology and innovation (STI) management.

I confirm that I have read this submission and believe that I have an appropriate level of expertise to confirm that it is of an acceptable scientific standard.

## References

[1] : Gender equity in scientific production in Peru and Colombia: reality or utopia in public universities?. *F1000Research* .2025;14: 10.12688/f1000research.170019.1 10.12688/f1000research.170019.1 40657179

# Reviewer report: Reviewer response for version 1

**DOI:** 10.5256/f1000research.187430.r425714

**Published:** 2025-10-31

**Authors:** Miguel Angulo-Giraldo

**Affiliations:** 1 Universidade Federal do Rio Grande do Sul (UFRGS), Lima, Peru

The analyzed text effectively raises a pertinent issue within the social sciences and provides an appropriate comparative framework. However, a crucial methodological aspect remains unresolved: the explicit criteria for selecting both countries for study. Although Peru and Colombia share a similar population size, the document lacks a robust justification regarding the comparability of their educational or scientific systems, a similarity that would be necessary to validate the comparison's underlying premises.

 Regarding the conclusions, they adequately meet the stated objective and align with the methodology employed. To significantly deepen the analytical contribution, I suggest a further exploration of the data:

1. **Disaggregation by Sex and Field of Knowledge:** The study's rigor would be enhanced by **disaggregating scientific output by the variable of "sex"** (since "gender" appears unavailable for study in this context) in relation to **broad disciplinary areas**. This analysis would reveal whether the observed phenomenon—for instance, production disparities—exhibits variation across different scientific fields.

This specific recommendation would transition the analysis from a general observation to a more nuanced, field-specific exploration, significantly increasing the study's academic depth and explanatory power.

Is the work clearly and accurately presented and does it cite the current literature?

Yes

If applicable, is the statistical analysis and its interpretation appropriate?

Yes

Are all the source data underlying the results available to ensure full reproducibility?

Yes

Is the study design appropriate and is the work technically sound?

Yes

Are the conclusions drawn adequately supported by the results?

Yes

Are sufficient details of methods and analysis provided to allow replication by others?

Yes

Reviewer Expertise:

Education, social science.

I confirm that I have read this submission and believe that I have an appropriate level of expertise to confirm that it is of an acceptable scientific standard.
